# Genome Sequence of the Acidophilic Iron Oxidizer *Ferrimicrobium acidiphilum* Strain T23^T^

**DOI:** 10.1128/genomeA.00383-15

**Published:** 2015-04-30

**Authors:** Sebastian Eisen, Anja Poehlein, D. Barrie Johnson, Rolf Daniel, Michael Schlömann, Martin Mühling

**Affiliations:** a Institute of Biological Sciences, TU Bergakademie Freiberg, Freiberg, Germany; b Genomic and Applied Microbiology & Göttingen Genomics Laboratory, Georg-August-Universität Göttingen, Göttingen, Germany; c College of Natural Sciences, Bangor University, Bangor, United Kingdom

## Abstract

Extremely acidophilic iron-oxidizing bacteria have largely been characterized for the phyla *Proteobacteria* and *Nitrospira*. Here, we report the draft genome of an iron-oxidizing and -reducing heterotrophic mesophile of the *Actinobacteria*, *Ferrimicrobium acidiphilum*, which was isolated from an abandoned pyrite mine. The genome sequence comprises 3.08 Mb.

## GENOME ANNOUNCEMENT

*Ferrimicrobium acidiphilum* strain T23^T^ is a Gram-positive acidophilic bacterium that was isolated from an enrichment culture inoculated with acidic water (pH 2.3) from an abandoned pyrite mine in north Wales (1). This bacterium, which both oxidizes and reduces iron, belongs to the subclass *Acidimicrobidae* within the *Actinobacteria*. The similarity of its 16S rRNA gene sequence compared to those of the type strains of the five recognized (*Acidimicrobium*, *Ferrithrix*, and *Aciditerrimonas*) or proposed members (*Acidithrix* and *Acidithiomicrobium*) of the *Acidimicrobidae* (2) ranges from 92% to 94%.

Chromosomal DNA of *F. acidiphilum* T23^T^ was isolated with the MasterPure complete DNA purification kit (Epicentre). The extracted DNA was used to generate 454-shotgun and Illumina-shotgun libraries, according to the manufacturers’ protocols. The libraries were sequenced using a 454 GS-FLX system (Titanium GS70 chemistry; Roche Life Science) and Genome Analyzer II (Illumina). Sequencing resulted in 52,582 454-shotgun and 3,993,736 paired-end Illumina reads, which corresponds to 12.2-fold and 145-fold estimated genome coverages, respectively. Assembly of the reads using the Roche Newbler assembly software 2.9 and MIRA (3) resulted in a total of 68 contigs. The draft genome sequence of *F. acidiphilum* T23^T^ comprises a genome of 3.08 Mb, with an overall G+C content of 55.3 mol%. Functional annotation of the 3,015 predicted protein-coding genes was initially carried out with the Integrated Microbial Genomes-Expert Review (IMG-ER) system (4). Subsequently, the annotations were manually curated using the Swiss-Prot, TrEMBL, and InterPro databases (5). The genome harbors 6 rRNA genes and 46 tRNA genes, which were identified with RNAmmer (6) and tRNAscan (7), respectively.

A detailed view on the protein-coding genes revealed that *F. acidiphilum* T23^T^ appears to utilize sulfate as its sole source of sulfur. Although phosphate can be stored in form of polyphosphate granules (8), and the uptake of inorganic phosphate is likely to be regulated by the Pho regulon (9), genes encoding proteins required for the cellular uptake of inorganic phosphate have not been detected in the genome.

Amino acids may be the sole nitrogen source for the strain, since the genome harbors 20 genes related to amino acid ABC-type transporters but lacks any nitrate, nitrite, or ammonium uptake mechanism and any nitrogenase-related genes. This, however, is in contradiction to the observed ability of *F. acidiphilum* T23^T^ to also grow in yeast extract (the only source of N)-free medium (1). The genome sequence analysis revealed the presence of both subunits of a type I RubisCO and of an additional 11 enzymes involved in CO_2_ fixation *via* the Calvin-Benson-Bassham cycle. In contrast to this observation, the ability of the strain to fix CO_2_ has, however, not been detected. In the context of CO_2_ fixation, it should further be noted that the genome appears to lack putative genes encoding both carboxysome shell proteins and a carbonic anhydrase.

### Nucleotide sequence accession numbers. 

The results from this genome sequencing project have been deposited at GenBank under the accession no. JXUW00000000. The version described in this paper is version JXUW01000000.
